# Clinical and outcome comparison of genetically positive vs. negative patients in a large cohort of suspected familial hypocalciuric hypercalcemia

**DOI:** 10.1007/s12020-023-03560-y

**Published:** 2023-10-30

**Authors:** Queralt Asla, Helena Sardà, Núria Seguí, Guillermo Martínez de Pinillos, Isabel Mazarico-Altisent, Ismael Capel, José Rives, Javier Suárez, Verónica Ávila-Rubio, Manuel Muñoz Torres, Ignasi Saigí, Nuria Palacios, Eulàlia Urgell, Susan M. Webb, Mercè Fernández, Josep Oriola, Mireia Mora, Mireia Tondo, Anna Aulinas

**Affiliations:** 1 Department of Endocrinology and Nutrition, Hospital de la Santa Creu i Sant Pau, Barcelona, Spain; 2 Sant Pau Biomedical Research Institute (IIB-Sant Pau), Hospital de la Santa Creu i Sant Pau, Barcelona, Spain; 3 Department of Medicine, University of Vic-Central University of Catalonia, Vic, Spain; 4 Department of Medicine, Universitat Autònoma de Barcelona, Bellaterra, Spain; 5 Department of Endocrinology and Nutrition, Hospital Clínic, Barcelona, Spain; 6 Department of Endocrinology, Hospital Universitario Virgen de Valme, Sevilla, Spain; 7 Department of Endocrinology and Nutrition, Hospital Universitari Parc Taulí, Sabadell, Barcelona Spain; 8 Institut d’Investigació i Innovació Parc Taulí (I3PT), Sabadell, Barcelona Spain; 9 Department of Biochemistry, Hospital de la Santa Creu i Sant Pau, Barcelona, Spain; 10 Cardiovascular Biochemistry, Biomedical Research Institute Sant Pau (IIB-Sant Pau), Barcelona, Spain; 11 Department of Endocrinology and Nutrition, Hospital Arnau de Vilanova, Lleida, Spain; 12 Department of Endocrinology and Nutrition, Hospital Universitario Clínico San Cecilio, Granada, Spain; 13 Instituto de Investigación Biosanitaria de Granada (ibs.GRANADA), 18014 Granada, Spain; 14 CIBER on Frailty and Healthy Aging (CIBERFES), Instituto de Salud Carlos III, 28029 Madrid, Spain; 15 Department of Endocrinology and Nutrition, Hospital Universitari de Vic, Vic, Spain; 16 Department of Endocrinology, Hospital Universitario Puerta de Hierro Majadahonda, Majadahonda, Madrid Spain; 17 Centro de Investigación Biomédica en Red de Enfermedades Raras (CIBER-ER, Unit 747), ISCIII, Madrid, Spain; 18 Department of Endocrinology and Nutrition, Hospital de la Santa Creu i Sant Pau - Hospital Dos de Maig, Barcelona, Spain; 19 Department of Biochemistry and Molecular Genetic, CDB, Hospital Clínic, Barcelona, Spain; 20 Institut d’Investigacions Biomèdiques Pi i Sunyer (IDIBAPS), Barcelona, Spain; 21 Department of Medicine, Faculty of Medicine and Health Sciences, University of Barcelona, Barcelona, Spain

**Keywords:** Familial hypocalciuric hypercalcemia (FHH), primary hyperparathyroidism (PHPT), calcium disorders, calcium-sensing receptor (CaSR), *CASR* gene, *CASR* mutations

## Abstract

**Objective:**

Biochemical suspicion of familial hypocalciuric hypercalcemia (FHH) might provide with a negative (FHH-negative) or positive (FHH-positive) genetic result. Understanding the differences between both groups may refine the identification of those with a positive genetic evaluation, aid management decisions and prospective surveillance. We aimed to compare FHH-positive and FHH-negative patients, and to identify predictive variables for FHH-positive cases.

**Design:**

Retrospective, national multi-centre study of patients with suspected FHH and genetic testing of the *CASR*, *AP2S1* and *GNA11* genes.

**Methods:**

Clinical, biochemical, radiological and treatment data were collected. We established a prediction model for the identification of FHH-positive cases by logistic regression analysis and area under the ROC curve (AUROC) was estimated.

**Results:**

We included 66 index cases, of which 30 (45.5%) had a pathogenic variant. FHH-positive cases were younger (*p* = 0.029), reported more frequently a positive family history (*p* < 0.001), presented higher magnesium (*p* < 0.001) and lower parathormone levels (*p* < 0.001) and were less often treated for hypercalcemia (*p* = 0.017) in comparison to FHH-negative cases. Magnesium levels showed the highest AUROC (0.825, 95%CI: 0.709–0.941). The multivariate analysis revealed that family history and magnesium levels were independent predictors of a positive genetic result. The predictive model showed an AUROC of 0.909 (95%CI: 0.826–0.991).

**Conclusions:**

The combination of magnesium and a positive family history offered a good diagnostic accuracy to predict a positive genetic result. Therefore, the inclusion of magnesium measurement in the routine evaluation of patients with suspected FHH might provide insight into the identification of a positive genetic result of any of the CaSR-related genes.

## Introduction

Hypercalcemia is often an incidental finding increasingly detected during routine blood tests in asymptomatic adult patients. An accurate differential diagnosis is necessary for its appropriate management [1]. One of the causes of hypercalcemia is familial hypocalciuric hypercalcemia (FHH).

FHH is an infrequent and lifelong disorder with an estimated prevalence range from 1:10.000 to 1:100.000, however, some studies suggest that it is underestimated [2–4]. FHH is a genetically heterogeneous disease due to heterozygous loss-of-function pathogenic variants of the calcium-sensing receptor (*CASR gene*) (FHH1 #145980) or its downstream regulatory pathway (*GNA11* and *AP2S1 genes*) (FHH2 #145981 and FHH3 #600740, respectively) [2]. FHH1 is the most frequent genotype, followed by FHH3, while FHH2 is extremely rare [2, 5, 6]. The hypoactivity of calcium-sensing receptor (CaSR) facilitates calcium (Ca^2+^) renal absorption and parathormone (PTH) production despite mildly elevated serum Ca^2+^ levels, resulting in different degrees of hypercalcemia, hypocalciuria and inappropriately elevated PTH [2].

FHH has an autosomal dominant inheritance pattern with a familial penetrance >90% [3]. Importantly, despite PTH levels are usually higher in PHPT than in FHH, the biochemical phenotype of FHH is sometimes difficult to distinguish from that of PHPT [7], except for urinary Ca^2+^ excretion which is typically low in FHH. A reduced urinary Ca^2+^ excretion, best expressed as 24-hour urine calcium-to-creatinine clearance ratio (CCCR), is commonly observed in FHH, and constitutes the main differential biochemical finding in FHH and PHPT. A CCCR > 0.02 virtually rules out FHH and is suggestive of PHPT, while a CCCR < 0.01 has a sensitivity of 65–80% and specificity of 74–88% to diagnose FHH [8–12]. However, values between 0.01 and 0.02 are helpless in the differential diagnosis. To date, a two-step diagnostic approach has been proposed, starting with CCCR screening and only performing genetic testing when CCCR is <0.02 [8]. However, up to 60% of the patients with CCCR < 0.02 eventually present PHPT and would require a genetic testing based on the traditional two-step diagnostic approach [8, 13, 14]. Moreover, genetic testing has limited sensitivity; more than 25% of patients with clinical and biochemical suspicion of FHH have a negative or uninformative genetic test result (referred to as genotype-negative) [10]. However, a negative result does not necessary exclude FHH and follow-up of these patients is recommended according to the recent European expert consensus of the ESE Educational Program of Parathyroid Disorders [10]. Collectively, these data suggest that performing genetic testing in all patients suspected of having FHH with a CCCR < 0.02 might not be either practical or cost-effective, given the biochemical overlap between both endocrine disorders (PHPT and FHH) and the low estimated prevalence of FHH.

Currently, it is still unclear if there are clinical, biochemical, and radiological differences between patients who strictly fulfil the biochemical suspicion criteria of FHH and are genetically positive vs. those genetically negative, that can be useful to better discriminate and accurately identify those patients with the highest probability for a positive genetic evaluation.

Thus, this study aimed at (1) improving the clinical characterization of patients with FHH-phenotype comparing clinical, biochemical, imaging data and therapeutic strategies of genetically positive FHH index cases (FHH-positive) and genetically negative patients (FHH-negative) and (2) identifying clinical, biochemical and/or radiological variables predictive for FHH-positive cases. Understanding the differences between FHH-positive and FHH-negative subjects might refine the identification of those patients with a positive genetic evaluation, and aid management decisions and follow-up in this group of patients.

## Patients and methods

### Patients

This is a retrospective, national multi-centre study of patients with clinical and biochemical suspicion of FHH in whom genetic testing for any of the loss-of-function variants of the *CASR* or its downstream regulatory pathway were performed.

The study protocol was approved by the coordinating centre (Hospital de la Santa Creu i Sant Pau) Institutional Review Board (EC/20/359/6149) and confirmed by the local Ethics Committee when legally required of the participating centres. Given the retrospective and descriptive character of the study, a waiver for informed consent was granted. All the data were pseudo anonymised. The study was preregistered at ClinicalTrials.gov (identifier NCT04872894).

From 2007 to 2022, we included all the patients with (1) a genetic test for the *CASR* (NM_000388.4), the *AP2S1* (NM_004069.4) and the *GNA11* (NM_002067.4) genes performed and (2) clinical and biochemical suspicion of FHH. Patients were referred from the Endocrinology Departments of 7 university and tertiary care hospitals (Hospital de la Santa Creu i Sant Pau – Barcelona, Hospital Clínic de Barcelona – Barcelona, Hospital Universitari Parc Taulí – Sabadell, Hospital Arnau de Vilanova – Lleida, Hospital Universitario Virgen de Valme – Sevilla, Hospital Universitario Clínico San Cecilio – Granada, and Hospital Universitario Puerta de Hierro Majadahonda – Majadahonda); and from the Endocrinology Department of one university and secondary care hospital (Hospital Universitari de Vic – Vic). Treating clinicians ordered the genetic evaluation to patients presenting with a biochemical profile suspicious of FHH, as well as to relatives of patients with a positive genetic result.

We considered a biochemical suspicion of FHH if the following criteria were met: (1) hypercalcemia defined as albumin-adjusted Ca^2+^ concentration ≥2.55 mmol/L, (2) elevated or inappropriately normal PTH concentrations according to each reference range and (3) reduced renal Ca^2+^ excretion assessed as CCCR < 0.02, despite appropriate serum 25-hydroxyvitamin D concentrations (>50 nmol/L). Exclusion criteria were: (1) absence of hypercalcemia, (2) CCCR ≥ 0.02, (3) none-index genetically positive (FHH-positive) patients, (4) insufficient available information and (5) any other known cause of hypercalcemia. Negative genetic testing (genetically negative) was defined as *CASR*, *AP2S1* or *GNA11* sequencing analysis with a negative result. Index cases were defined as the first diagnosed FHH case in a kindred.

### Data collection

The clinical, biochemical, radiological and therapeutic data were retrospectively collected from the clinical files at each participating centre. A specific dataset was designed including the following items:

1. Demographical and baseline characteristics: sex, age at diagnosis of first elevated serum Ca^2+^ levels, age at genetic evaluation, family history.
2. Clinical characteristics and comorbidities associated to hypercalcemia: kidney stones, bone mineral density (osteopenia, osteoporosis) assessed by a Dual Energy X-ray Absorptiometry scan when available, history of fragility bone fractures, pancreatitis, cardiovascular disease and neuropsychiatric disease.
3. Biochemical data at the time of diagnosis of hypercalcemia and being treatment-naïve for hypercalcemia: serum albumin-adjusted Ca^2+^ (mmol/L), PTH (pmol/L), phosphate (mmol/L), magnesium (Mg^2+^) (mmol/L), 25-hydroxivitamin D concentrations (nmol/L), estimated glomerular filtration rate (eGFR) and CCCR.
4. Genetic data: genes evaluated (*CASR*, *GNA11*, *AP2S1* and *MEN1*), results of genetic testing (positive vs. negative) and the reported pathogenic variant.
5. Imaging characteristics: results of neck ultrasound, 99 m Tc-sestamibi parathyroid scintigraphy, 18^F^-Choline positron emission tomography and a computed tomography (PET/CT), or neck computed tomography (CT).
6. Therapeutic strategies for hypercalcemia: observation, hydration, pharmacological treatment or surgery.

### Biochemical measurements

Fasting blood samples were collected and the following parameters were measured in serum according to standard commercially available assays: Ca^2+^, PTH, Mg^2+^, phosphate, albumin, creatinine and 25-hydroxyvitamin D; additionally, 24-h urinary Ca^2+^ and creatinine were measured. We calculated the eGFR (mL/min/1.73m^2^) using the MDRD (Modification of Diet in Renal Disease) formula, the albumin-adjusted Ca^2+^ concentration (mmol/L) as total calcemia (mmol/L) – 0.025 * (serum albumin (g/L) - 40), and the renal calcium/creatinine clearance ratio (CCCR) as (24-h urine Ca^2+^/total serum Ca^2+^)/(24-h urine creatinine/serum creatinine).

### Gene amplification and sequencing

Genomic DNA was isolated from whole blood using the QIAamp DNA blood minikit (Qiagen, Hilden, Germany). All the coding-exons and exon-flanking intronic regions of the *CASR, AP2S1*, and *GNA11* genes were amplified by PCR. The resulting products were purified using GFX PCR DNA and a Gel Band Purification Kit (GE Healthcare, Buckinghamshire, UK) and sequenced using a Big Dye Terminator cycle sequencing kit v.3.1 (Applied Biosystems, Foster, CA, USA) on an ABI3130XL automated analyzer (Applied Biosystems). The resulting chromatograms were analysed with the Staden package program [15]. The primers used for gene amplification and Sanger sequencing are available upon request. A sequential analysis was performed to optimize the diagnostic process.

### Characterization of variants and bioinformatics analysis

The nomenclature of the allelic variants follows the recommendations of the Human Molecular Genome Variation Society (http://www.hgvs.org). To characterize the variants, they were checked with the Human Gene Mutation Database (HGMD, www.hgmd.cf.ac.uk), GnomAD (https://gnomad.broadinstitute.org/), and ClinVar (www.ncbi.nlm.nih.gov/clinvar) databases. Bioinformatics functional analysis was also used. The impact of point mutations on the protein was assessed with the following software: SIFT (sift.bii.a-star.edu.sg) [16], PolyPhen2 (genetics.bwh.harvard.edu/pph2/index.shtml) [17], Provean (provean.jcvi.org) [18], and Mutation Taster (http://www.mutationtaster.org) [19]. Point mutations causing a premature stop codons, small insertions or deletions causing a frameshift and a premature stop codon, large rearrangements, and mutations affecting intron donor or acceptor splice sites were considered pathogenic [20]. The remaining variants were considered pathogenic depending on the existence of functional analysis previously reported in the literature, identification as pathogenic or likely pathogenic in databases such as ClinVar, or in the absence of previous information, when the programs used in the bioinformatics analysis gave as a result probable alteration of the protein function.

### Statistical analysis

We presented discrete variables as frequency (percentage) and continuous variables as means with standard error of the mean (SEM) or as medians and interquartile ranges (p25-p75), as appropriate. We assessed intergroup comparisons (FHH-negative vs. FHH-positive) applying the Fisher’s exact test, Student t-test or Wilcoxon test, as appropriate.

We tested predictive efficacy of the most relevant variables to discriminate patients as FHH-positive vs. FHH-negative using the area under the receiver operating characteristic (AUROC) curve (roctab and rocreg Stata functions). We calculated the optimal cut-off values using the Youden index method. We used a multivariant logistic regression model to test the association between clinically relevant variables and/or those significantly different in the univariate analyses (across FHH positive vs. FHH negative) and FHH-genotype. The estimated adjusted odds ratios (OR) and their 95% confidence intervals (95%CI) were reported. The model discriminative performance and accuracy was tested by Hosmer-Lemeshow goodness-of-fit test and AUROC curve. We reported calculated AUROC curve and its 95%CI.

We performed statistical analysis using STATA software, version 14.2 (StataCorp LLC, College Station, TX). All *p*-values were two-sided, and significance was set at *p* < 0.05.

## Results

### Baseline characteristics and comorbidities associated with hypercalcemia

A total of 92 patients with clinical and biochemical suspicion of FHH and a genetic test result available were included in the database. Twenty-six patients were excluded: 15 were not index cases, 7 had a variant of uncertain significance in the genetic evaluation and 4 did not have critical information available. Finally, 66 cases were eligible, of which 36 (54.5%) were FHH-negative and 30 (45.5%) patients FHH-positive.

Table 1 summarizes the baseline characteristics and phenotypic differences between FHH-genotype. FHH-positive in comparison to FHH-negative patients, were younger at diagnosis (*p* = 0.029), reported more frequently a family history of hypercalcemia (*p* < 0.001), and had a lower frequency of kidney stones (*p* = 0.010). No other differences in clinical characteristics and comorbidities were observed among groups.

**Table 1:** Summary of demographic and baseline characteristics, and medical comorbidities associated to hypercalcemia according to FHH-genotype

	FHH-negative (*n* = 36, 54.5%)	FHH-positive (*n* = 30, 45.5%)	*p*
Demographical and baseline characteristics			
Sex (Male/Female), *n* (%)	18 (50%)/18 (50%)	9 (30%)/21 (70%)	0.13
Age at diagnosis (years)	67.4 (23.9)	56.1 (24.6)	0.03
Time elapsed from hypercalcemia diagnosis to genetic study (years)	3.4 (6.8)	5.7 (8.7)	0.06
Family history, *n* (%)	8 (22%)	20 (67%)	<0.001
Medical comorbidities			
Kidney stones, *n* (%)	14 (39%)	3 (10%)	0.01
Diagnosis of osteopenia, *n* (%)	15 (42%)	8 (27%)	0.29
Diagnosis of osteoporosis, *n* (%)	11 (31%)	6 (20%)	0.41
Fragility fractures, *n* (%)	3 (8%)	0 (0%)	0.24
History of pancreatitis, *n* (%)	1 (3%)	1 (3%)	1.00
Prevalence of cardiovascular disease, *n* (%)	17 (47%)	9 (30%)	0.21
Neuropsychiatric disease, *n* (%)	6 (17%)	3 (10%)	0.49

Data are reported as median (p25-p75) (non-Gaussian distribution)

*FHH* familial hypocalciuric hypercalcemia

### Genetic characteristics

Sequencing of the *CASR* and *AP2S1* genes were performed in all included participants, whereas *GNA11* gene was studied in 34.8% (23/66) of participants. *MEN1* gene had been previously sequenced in 27.3% (18/66) of the participants.

We identified twenty-one pathogenic variants in patients presenting with a clinical and biochemical profile suspicious of FHH (Table 2). Of those, 43.9% (29 of 66) were in the *CASR* gene and 1.5% (1/66) in the *AP2S1* gene. No pathogenic variants were found in *GNA11* and *MEN1* genes.

**Table 2:** Pathogenic variant description of the FHH-positive participants (*n* = 30)

N° of patients affected	Gene	Nomenclature DNA	Nomenclature protein	State in the literature
1	*AP2S1*	c.43C > T	p.(Arg15Cys)	Already reported
1	*CASR*	c.107G > A	p.(Gly36Glu)	Not described
7	*CASR*	c.164C > T	p.(Pro55Leu)	Already reported
1	*CASR*	c.413C > T	p.(Thr138Met)	Already reported
1	*CASR*	c.473G > C	p.(Gly158Ala)	Not described
1	*CASR*	c.491A > G	p.(Gln164Arg)	Already reported
1	*CASR*	c.492+1G > A	NA	Not described
2	*CASR*	c.554G > A	p.(Arg185Gln)	Already reported
1	*CASR*	c.659G > A	p.(Arg220Gln)	Already reported
1	*CASR*	c.1394G > A	p.(Arg465Gln)	Already reported
1	*CASR*	c.1636T>G	p.(Cys546Gly)	Already reported
1	*CASR*	c.2039G > A	p.(Arg680His)	Already reported
1	*CASR*	c.2089G > A	p.(Val697Met)	Already reported
1	*CASR*	c.2101C > G	p.(Arg701Gly)	Already reported
1	*CASR*	c.2393C > T	p.(Pro798Leu)	Already reported
2	*CASR*	c.2411C > A	p.(Ala804Asp)	Already reported
2	*CASR*	c.2485del	p.(Tyr829Metfster8)	Not described
1	*CASR*	c.2525T > C	p.(Leu842Pro)	Not described
2	*CASR*	c.2656C > G	p.(Arg886Gly)	Not described
1	*CASR*	c.3236A > C	p.(Ter1079Serext*8)	Not described

*NA* not applicable

### Biochemical and imaging characteristics

**Fig. 1:**
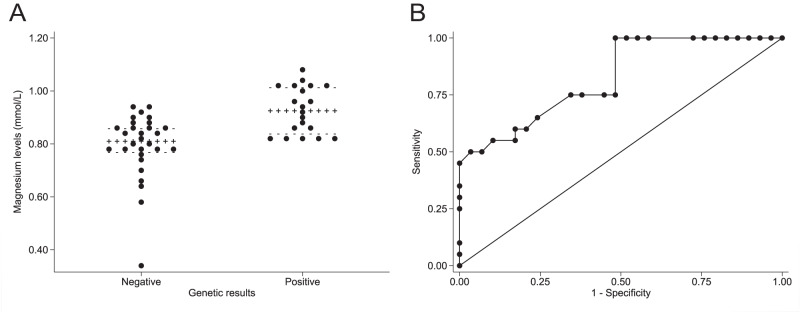
Serum Mg^2+^ levels. **A** Serum Mg^2+^ levels according to FHH-genotype. Serum Mg^2+^ levels are lower in the FHH-negative than in the FHH-positive group (*p* < 0.001). **B** Discriminative accuracy of serum Mg^2+^ levels for classifying patients as FHH-negative or FHH-positive. The area under the ROC curve was 0.825 (95% CI: 0.709–0.941)

Table 3 summarizes the main biochemical and imaging characteristics of the study participants. FHH-negative in comparison to FHH-positive participants, had higher PTH levels (*p* < 0.001), but lower serum Mg^2+^ levels (*p* < 0.001) (Fig. 1a). Although eGFR was >60 mL/min/1.73m^2^, mean eGFR was slightly higher in the FHH-positive than in the FHH-negative group (*p* = 0.039).

**Table 3:** Biochemical parameters at diagnosis and imaging characteristics of the study participants according to FHH-genotype

	FHH-negative (*n* = 36, 54.5%)	FHH-positive (*n* = 30, 45.5%)	Reference range	*P*
Biochemical parameters at diagnosis				
Serum Ca^2+^ (mmol/L)	2.64 (0.24)	2.73 (0.21)	2.10–2.55	0.24
Highest serum Ca^2+^ (mmol/L)	2.78 (0.32)	2.86 (0.25)		0.43
Serum PTH (pmol/L)	13.18 (11.36)	6.50 (5.77)	1.60–6.90	<0.001
Serum phosphate (mmol/L)	0.84 (0.26)	0.91 (0.26)	0.87–1.45	0.20
Serum Mg^2+^ (mmol/L)*	0.81 (0.09)	0.93 (0.18)	0.66–1.07	<0.001
eGFR (mL/min/1.73m^2^)	70.76 ± 3.81	84.42 ± 3.92		0.04
25-hydroxivitamin D (nmol/L)	51.02 (50.36)	49.00 (36.9)	>50	0.53
CCCR ratio	0.009 ± 0.001	0.009 ± 0.001		0.68
Imaging characteristics				
99 m Tc-sestamibi parathyroid scintigraphy (done), *n* (%)	34 (94%)	21 (70%)		0.02
Findings				
Normal, *n* (%)	20 (59%)	19 (90%)		0.03
Uniglandular involvement, *n* (%)	12 (35%)	2 (10%)		
Multiglandular involvement, *n* (%)	2 (6%)	0 (0%)		
Neck ultrasound (done), *n* (%)	25 (69%)	21 (70%)		0.96
Findings				
Normal, *n* (%)	13 (52%)	19 (90%)		0.01
Uniglandular involvement, *n* (%)	12 (48%)	2 (10%)		
Neck CT scan (done), *n* (%)	12 (33%)	9 (30%)		0.79
Findings				
Normal, *n* (%)	10 (83%)	7 (78%)		0.75
Uniglandular involvement, *n* (%)	2 (17%)	1 (11%)		
Multiglandular, *n* (%)	0 (0%)	1 (11%)		
18^F^-Choline PET/CT (done), *n* (%)**	6 (17%)	0 (0%)		0.03
Findings				
Negative result, *n* (%)	0 (0%)	-		
Uniglandular involvement, *n* (%)	5 (83%)	-		
Multiglandular involvement, *n* (%)	1 (17%)	-		

Data are reported as mean ± standard error of the mean (Gaussian distribution) and as median (p25-p75) (non-Gaussian distribution)

*FHH* familial hypocalciuric hypercalcemia, *Ca*^2+^, albumin-adjusted calcium, *PTH* parathormone, *Mg*^2+^ magnesium, *eGFR* estimated glomerular filtration rate, *CCCR* calcium creatinine clearance ratio, *CT* computed tomography, *PET/CT* positron emission tomography/computed tomography

*Available in 49 participants (29 FHH-negative and 20 FHH-positive) ^**^Only available in two centres after 2016

99 m Tc-sestamibi parathyroid scintigraphy and neck ultrasound were the two most frequently employed imaging techniques (83% and 70% of the participants, respectively). Both scintigraphy and 18^F^-Choline PET/CT were more frequently used in FHH-negative participants, yet 18^F^-Choline PET/CT was only available in two centres at the time of data collection.

### Therapeutic management

Table 4 summarizes the therapeutic management across groups. Hydration and/or observation was the strategy of choice in most of the patients. Overall, FHH-negative patients received more frequently some kind of treatment for hypercalcemia than FHH-positive patients (75% vs. 44%, respectively, *p* = 0.017). Cinacalcet was more often used in FHH-negative patients (33% vs. 13%, *p* = 0.08).

**Table 4:** Therapeutic management of chronic hypercalcemia according to FHH-genotype

Therapeutic management	FHH-negative (*n* = 36, 54.5%)	FHH-positive (*n* = 30, 45.5%)	*p*
Hydration, n (%)	20 (56%)	14 (47%)	0.62
Diuretics, n (%)	5 (14%)	2 (7%)	0.44
Bisphosphonates, n (%)	6 (17%)	2 (7%)	0.28
Cinacalcet, n (%)	12 (33%)	4 (13%)	0.08
Parathyroid surgery, n (%)	16 (44%)	8 (27%)	0.20
Type of parathyroid surgery, n (%)			0.75
Uniglandular, n (%)	9 (56%)	5 (63%)	
Subtotal, n (%)	5 (31%)	3 (38%)	
Total, n (%)	1 (6%)	0 (0%)	

*FHH* familial hypocalciuric hypercalcemia

Parathyroid surgery was performed in 24 patients (16 FHH-negative and 8 FHH-positive, *p* = 0.199), being uniglandular parathyroidectomy the most frequent type of surgery. Pathological reports significantly differed among groups. Parathyroid adenoma was found in 12 of 16 (75%) of FHH-negative operated patients and in 1 of 8 (13%) of FHH-positive operated patients, while parathyroid hyperplasia was more common in FHH-positive cases (9% vs. 13% respectively) (*p* = 0.015). Normalization of serum Ca^2+^ concentrations were observed in 13 of 15 (87%) FHH-negative operated patients and in 2 of 7 (29%) FHH-positive operated patients (*p* = 0.014). Serum Ca^2+^ concentrations were not available in two patients after surgery.

### Predictive criteria for the identification of loss-of-function variants in FHH

We performed AUROC curve analyses to identify the variable that anticipated with highest accuracy a positive genetic result. Serum Mg^2+^ levels showed the highest AUROC curve (0.825, 95%CI: 0.709–0.941) (Fig. 1b), followed by serum PTH levels (0.744, 95%CI: 0.616–0.873) and a positive family history (0.722, 95%CI: 0.612–0.832). The optimal cut-off point of Mg^2+^ levels for a correct classification of a participant as FHH-negative or FHH-positive was 0.82 mmol/L, yielding a sensitivity of 100% and a specificity of 52% and classifying correctly 72% of the patients. Specifically, none of the FHH-positive participants presented with serum Mg^2+^ levels <0.82 mmol/L and none of the FHH-negative participants presented with serum Mg^2+^ levels >0.94 mmol/L, despite substantial overlap between groups (Fig. 1a).

We performed a multivariant logistic analysis to identify predictive criteria for any of the loss-of-function variants of the calcium-sensing genes including those variables clinically relevant or significant in the bivariate analysis (family history, age, and serum PTH and Mg^2+^ levels). Logistic regression analysis revealed that family history (OR 14.6, 95%CI [1.59.-134], *p* = 0.018) and serum Mg^2+^ levels (OR 11.38 for each tenth increment of Mg^2+^ levels, 95%CI [2.27–57.11], *p* = 0.003) were independent predictors of FHH-positive regardless of serum PTH levels (OR 0.31, 95%CI [0.10–1.08], *p* = 0.068) and age (OR 0.76, 95%CI [0.03–17.30], *p* = 0.865). The Hosmer-Lemeshow test (χ^2^ = 3.7, *p* = 0.883) showed a good degree of fit.

**Fig. 2:**
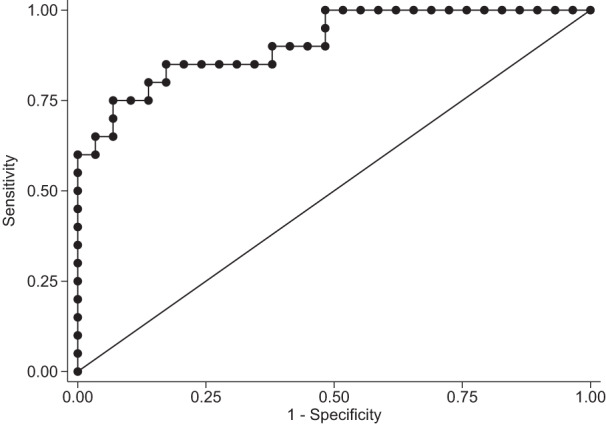
The area under the ROC curve of the model for the discrimination between FHH-positive and FHH-negative participants was 0.909 (95%CI: 0.826–0.991). Footnote: the model included the following variables: family history, serum Mg^2+^ levels, PTH levels and age

The AUROC curve of the model was 0.909 (95% CI: 0.826 to 0.991) (Fig. 2). The optimal cut-off of the model yielded a diagnostic sensitivity of 75.0%, specificity of 93.1% and correctly classified 85.71% of the study participants, either FHH-negative or FHH-positive.

## Discussion

Herein we describe a large cohort of patients with suspected FHH in whom a genetic evaluation was performed. Since it has traditionally been accepted that CCCR plays a key role in distinguishing PHPT from FHH [8, 10, 14], we only included patients who fulfilled strict biochemical criteria suspicious for FHH based on CCCR. By observing in our clinical practice that almost half of the patients had a negative genetic result despite the biochemical suspicion of FHH, we pursued a comparative evaluation of clinical, biochemical, imaging and therapeutic strategies between FHH-negative and FHH-positive cases to better refine the identification of those individuals who might have a positive genetic evaluation. To our knowledge this is the largest cohort of patients with suspected FHH reported in Spain. FHH-positive patients had more frequently a positive family history, a lower prevalence of kidney stones, lower serum PTH levels, higher serum Mg^2+^ levels and were less often treated for hypercalcemia. Remarkably, the multivariate analysis revealed that the combination of serum Mg^2+^ levels together with a positive family history provided a high accuracy for identifying those participants with the highest probability for a positive genetic result. Ultimately, our preliminary data highlight the importance of measuring routinely Mg^2+^ levels for the evaluation of hypercalcemia.

Mg^2+^ is a cation whose renal absorption occurs in the same location as that of Ca^2+^, at the loop of Henle where the *CASR* is mainly expressed, and it is controlled by several hormonal and nonhormonal factors, including PTH and CaSR, respectively [6, 21, 22]. One explanation could be that the hypoactivity of CaSR, due to loss-of-function pathogenic variants of *CASR*, facilitates not only Ca^2+^ renal absorption but also Mg^2+^ renal absorption [21], resulting in higher serum Mg^2+^ levels compared to normal CaSR activity. In the same line of findings, previous studies reported higher serum Mg^2+^ levels in patients diagnosed of FHH when compared to PHPT [6, 22, 23]. In particular, we observed that serum Mg^2+^ levels >0.82 mmol/L provided the maximum sensitivity for a positive genetic result, but with a modest specificity. Interestingly, the multivariate analysis showed that the combination of serum Mg^2+^ levels and family history correctly classified 86% of the patients as FHH-positive or FHH-negative. Interestingly, a risk prediction tool named Pro-FHH including serum Ca^2+^ levels, PTH, osteocalcin and CCCR has been proposed to better discriminate between FHH and PHPT [23]. Our preliminary observations suggest that a risk prediction tool incorporating serum Mg^2+^ levels and the family history could be useful to distinguish between FHH-negative vs. FHH-positive patients and to identify those with the highest probability for a positive genetic result related to their FHH biochemical phenotype. However, a larger and prospective study is needed to confirm our findings and to develop a risk prediction tool to discriminate between FHH-negative and FHH-positive cases that could be used in clinical practice.

We detected pathogenic variants in half of the patients with a suspected biochemical phenotype of FHH and the distribution of pathogenic variants observed was similar to that described in the literature [2, 5, 6]. Mariathasan et al found that family history was the strongest predictor for the presence of a hereditary form of PHPT or FHH in a large UK cohort [12]. Due to its high familiar penetrance (>90%) [3], the family history is a key feature in the evaluation of FHH and should be deeply interrogated in all cases with a biochemical phenotype suspicious of FHH. Intriguingly though, in our cohort up to 27% of FHH-positive patients were operated as a result of symptomatic and/or high serum Ca^2+^ concentrations before any genetic evaluation that was performed later on when hypercalcemia recurred after surgery.

Estimated GFR was normal in all included patients, however, was lower in the FHH-negative group. This finding was probably related to the higher prevalence of kidney stones in the FHH-negative group, despite similar serum Ca^2+^ levels and CCCR among groups. One explanation could be the existence of unrecognized parathyroid adenomas and consequently, the presence of PHPT in the FHH-negative group. Along these lines a recent study reported up to 17% of patients with PHPT with a CCCR < 0.01 [9]. In our cohort, 75% of FHH-negative and 13% of FHH-positive patients, who underwent a parathyroid surgery, were eventually diagnosed with a parathyroid adenoma. Altogether these data suggest that a biochemical and clinical overlap between FHH-negative, FHH-positive and PHPT cases might exist. On the one hand, the concomitant occurrence of FHH-positive patients with a parathyroid adenoma, although extremely infrequent, is possible in the same patient [24, 25], and on the other hand, a significant proportion of patients with a clear FHH phenotype the genetic evaluation can be negative without any concomitant parathyroid adenoma. Intriguingly, two patients with confirmed FHH who underwent surgery, became unexpectedly normocalcemic after surgery. It might be possible that albumin-adjusted calcium levels are not accurate enough to identify mild hypercalcemia. Despite ongoing discussion about which calcium to measure, ionized calcium could be more precise in some cases [10].

The role of the CaSR in the regulation of Ca^2+^ homoeostasis is well established [26]. Commonly, heterozygous inactivating pathogenic variants of the *CASR* gene lead to FHH, whilst homozygous or compound heterozygous inactivating mutations cause severe neonatal hyperparathyroidism [2]. Apart from human disorders related to germline inactivating mutations of the *CASR* gene, over the last decades efforts have been made to demonstrate the role of somatic abnormalities of the *CASR* gene in PHPT [26, 27]. On the basis of a complex pathophysiology of PHPT, some authors suggest that FHH may be an atypical form of PHPT given the sharing of mutual features between both entities [24, 25, 27]. It has been proposed that over secretion of PTH in patients with PHPT is, among others, produced by an alteration of the CaSR set-point [28–30], being the immunohistochemical expression of the CaSR and CaSR mRNA expression reduced in parathyroid adenomas [29–31]. Collectively these data do not rule out the participation of the CaSR in parathyroid tumorigenesis. Actually, these data could partially explain the coexistence in a same patient of a germline *CASR* loss-of-function pathogenic variant and a parathyroid adenoma and the consideration of FHH-negative subjects as an atypical form of PHPT.

Several limitations need to be acknowledged. Due to the retrospective and multicentre character of the study, some data are missing, for instance, bone remodelling markers that were mostly not available. Nevertheless, we collected a considerable number of cases of this rare endocrine disease that fulfilled strict criteria of biochemical suspicion of FHH that allowed us to compare for the first time clinical, biochemical, imaging and therapeutic variables of FHH-negative versus FHH-positive patients. In addition, not all negative-*CASR* and negative-*AP2S1* gene pathogenic variants underwent further genetic study, so, although the prevalence of FHH3 is extremely rare, a few FHH-negative patients could be misclassified. However, in our cohort, the distribution of positive genetic results was similar to that described in the medical literature.

In conclusion, the combination of serum Mg^2+^ and a positive family history offered a good diagnostic accuracy to predict a positive genetic result. The inclusion of serum Mg^2+^ measurement in the routine evaluation of patients presenting with hypercalcemia and low urine CCCR might arouse suspicion of a positive genetic result of any of the CaSR-related genes.

### Supplementary Information

STROBE_checklist
